# 2041 The cell-cell adhesion component PLEKHA7 regulates the pro-tumorigenic MIR17HG long non-coding RNA in colon epithelial cells

**DOI:** 10.1017/cts.2018.129

**Published:** 2018-11-21

**Authors:** Mary C. Bridges, Joyce Nair-Menon, Antonis Kourtidis

**Affiliations:** 1 Medical University of South Carolina; 2 Department of Regenerative Medicine and Cell Biology, MUSC

## Abstract

OBJECTIVES/SPECIFIC AIMS: The goal of this study is to test the hypothesis that the adherens junctions of colon epithelial cells regulate lncRNAs levels and function via the microprocessor and RISC complexes to suppress expression of pro-tumorigenic markers and aberrant cell behavior. METHODS/STUDY POPULATION: To test this hypothesis, we used colon epithelial cancer cell lines. We performed RNA-seq following knockdown of PLEKHA7, a key component of the adherens junctions, to identify changes in lncRNA expression and downstream mRNA levels. We confirmed junctional localization of affected lncRNAs from the RNA-seq and those that we found in our preliminary study by using in situ hybridization (ISH). RESULTS/ANTICIPATED RESULTS: RNA-seq identified junction-associated lncRNAs whose expression levels are regulated by PLEKHA7. The top upregulated lncRNA upon PLEKHA7 depletion was MIR17HG, an oncogenic host transcript of a cluster of miRNAs. These mature miRNAs also co-precipitate with PLEKHA7. PLEKHA7 knockdown results in increased levels of MIR17HG, but only a subset of its hosted miRNAs (miR-19a,b). Notably, miR-19a and mir-19b are highly upregulated in colon cancer. Our data suggest that 2 PLEKHA7-associated miRNAs, miR-203a and miR-372, mediate suppression MIR17HG. Re-expression of PLEKHA7 in aggressive colon cancer cells that lack PLEKHA7 suppressed expression of MIR17HG, as well as anchorage independent growth of these cells. DISCUSSION/SIGNIFICANCE OF IMPACT: Our data point towards a novel mechanism of lncRNA regulation that tethers epithelial tissue integrity with pro-tumorigenic cell transformation. Reducing elevated MIR17HG levels, is a potential therapeutic approach to suppress the tumorigenic behavior of cells that have lost their junctional integrity and homeostasis. identify a network of miRNA-mRNA-lncRNA interactions that could be exploited for further mechanistic studies, as well as for diagnostic and therapeutic purposes in the future.

